# Genetic Diversity of the Critically Endangered *Thuja sutchuenensis* Revealed by ISSR Markers and the Implications for Conservation

**DOI:** 10.3390/ijms140714860

**Published:** 2013-07-16

**Authors:** Jianfeng Liu, Shengqing Shi, Ermei Chang, Wenjuan Yang, Zeping Jiang

**Affiliations:** State Key Lab of Tree Genetics and Breeding, Research Institute of Forestry, Chinese Academy of Forestry, Beijing 100091, China; E-Mails: liujf@caf.ac.cn (J.L.); shi.shengqing@hotmail.com (S.S.); changcem@gmail.com (E.C.); yangwenjuan31@126.com (W.Y.)

**Keywords:** *Thuja sutchuenensis*, genetic variation, ISSR markers, conservation implications

## Abstract

*Thuja sutchuenensis* Franch. is a critically endangered plant endemic to the North-East Chongqing, China. Genetic variation was studied to assess the distribution of genetic diversity within and among seven populations from the single remnant locations, using inter-simple sequence repeat (ISSR) markers. A total of 15 primers generated 310 well defined bands, with an average of 20.7 bands per primer. The seven populations revealed a relatively high level of genetic diversity in the species. The percentage of polymorphic bands, Nei’s gene diversity and Shannon’s information index at the population and species level were 76.1%, 0.155, 0.252 and 100%, 0.165, 0.295, respectively. A low level of genetic differentiation among populations (*G_ST_* = 0.102), in line with the results of Analyses of Molecular Variance (AMOVA), and a high level of gene flow (*N_m_* = 4.407) were observed. Both the Unweighted Pair Group Method with Arithmatic Mean (UPGMA) cluster analysis and Principal Coordinates Analysis (PCoA) supported the grouping of all seven populations into two groups. In addition, Mantel test revealed no significant correlation between genetic and geographical distances (*r* = 0.329, *p* = 0.100). The low genetic differentiation among populations implies that the conservation efforts should aim to preserve all the extant populations of this endangered species.

## 1. Introduction

*Thuja sutchuenensis* Franch., an evergreen coniferous species of genus *Thuja* in the cypress family Cupressaceae, is a critically endangered species endemic to mountain area in North-East Chongqing Municipality, China [1]. It was first described in 1899 from specimens collected by the French botanist Paul Guillaume Farges in 1892 and 1900, but was not seen again thereafter, despite many searches, for almost 100 years and was presumed to be extinct due to over-cutting for its valuable scented wood. A small number of specimens were rediscovered in 1999, growing on almost inaccessible steep ridges close to (or at the same site) where Farges had first found it [2]. The area of its occurrence has now been designated two Special Protection Area in order to protect the species. However, limited recruitment in remnant populations of the species was observed according to our continuous field investigation over 10 years.

Genetic variation within and among natural populations is crucial for the long-term survival of a species. Especially for rare or endangered species, an accurate estimate of the genetic variation among or within its populations could be helpful to address its endangered status or mechanism [3–6], and provide fundamental information in designing conservation programs [7]. Among various molecular tools for genetic analysis, inter-simple sequence repeats (ISSR), which involve PCR amplifications of DNA using a primer composed of a microsatellite sequence anchored at 3′ or 5′ end by 2–4 arbitrary nucleotides, are a powerful tool for investigating genetic variation within species [8–10]. Because of the higher annealing temperature and longer sequence of ISSR primers, they can yield more reliable and reproducible bands than Random Amplified Polymorphic DNA (RAPD) [11–13], and the cost of the analyses is relatively lower comparing to other markers such as Restriction Fragment Length Polymorphism (RFLP), Simple Sequence Repeat (SSR) and Amplified Fragment Length Polymorphism (AFLP) [14,15]. In addition, it does not require previous genomic sequence information, which makes ISSR technically simpler than many other marker systems [16]. Therefore, ISSR has been widely used for population genetic analysis of various plant species, including many rare or endemic plants, *Neopicrorhiza scrophulariiflora* [17], *Swertia tetraptera* [18], *Michelia coriacea* [19], *etc.*

In the present study, we used ISSR markers to investigate the genetic composition of natural *T. sutchuenensis* populations, which restrictedly distributed in North-East Chongqing, China, with the following aims: (1) to evaluate the genetic diversity at the population and species levels in the *T. sutchuenensis*; (2) to assess the distribution of the genetic variation within and among populations and to construct a dendrogram demonstrating the genetic relationships among them; and (3) to use the genetic information as a tool for assessing the current conservation management plan for this endangered species and for designing conservation strategies.

## 2. Results

### 2.1. Genetic Diversity

**Figure 1:**
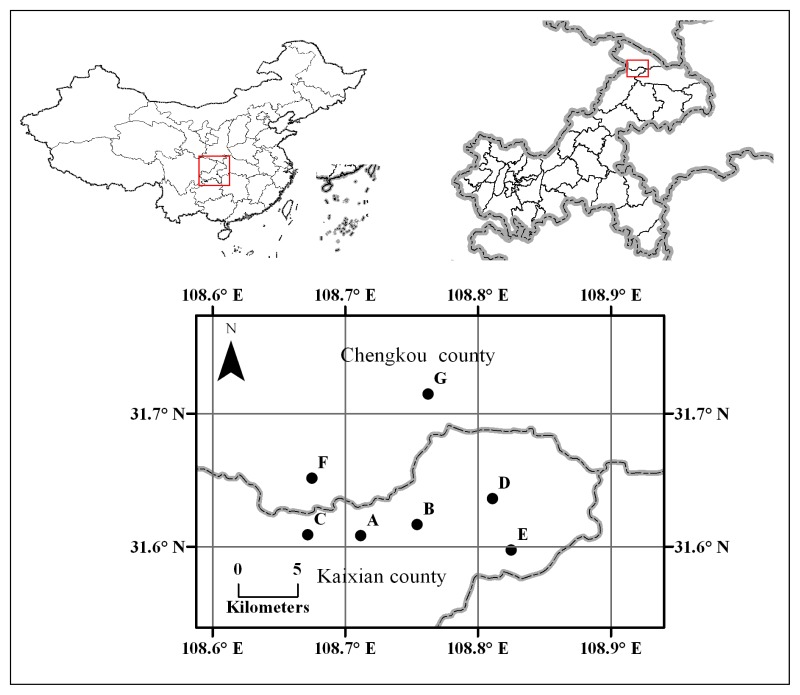
Geographic distribution of seven populations of *T. sutchuenensis* sampled for inter-simple sequence repeats (ISSR) analysis.

Fifteen selected ISSR primers were used to amplify all 139 DNA samples from seven natural populations of *T. sutchuenensis* (Table 1, Figure 1), and yielded 310 bright and discernible products in the range 130–950 bp (Table 2). At the population level, the percentage of polymorphic bands (*P*) ranged from 59.7% (A) to 91.3% (C) (Table 3) with an average of 76.1%, while with 100.0% at species level. The mean observed number of alleles (*N_a_*) ranged from 1.597 to 1.913, while the mean effective number of alleles (*N_e_*) varied from 1.180 to 1.237. Nei’s genetic diversity (*H*) varied from 0.130 to 0.178, with an average of 0.155, and Shannon’s information indices (*I*) ranged from 0.180 to 0.291, with an average of 0.252. At species level, *H* and *I* were 0.165 and 0.295, respectively.

### 2.2. Genetic Differentiation

The total genetic diversity (*H_T_*) of the species and genetic diversity within populations (*H_S_*) were 0.167 and 0.150, respectively (Table 3). The proportion of genetic variation contributed by the differences among populations (*G_st_*) was 0.102, thus leaving 89.8% of the total genetic variation harboured within the populations. It was consistent with the results of AMOVA, which detected highest genetic variation within population (87.9%), whereas the variance among populations was only 12.1% (Table 4). This was further confirmed by the abundant gene flow among populations (*N_m_* = 4.407).

### 2.3. Cluster Analysis

**Figure 2:**
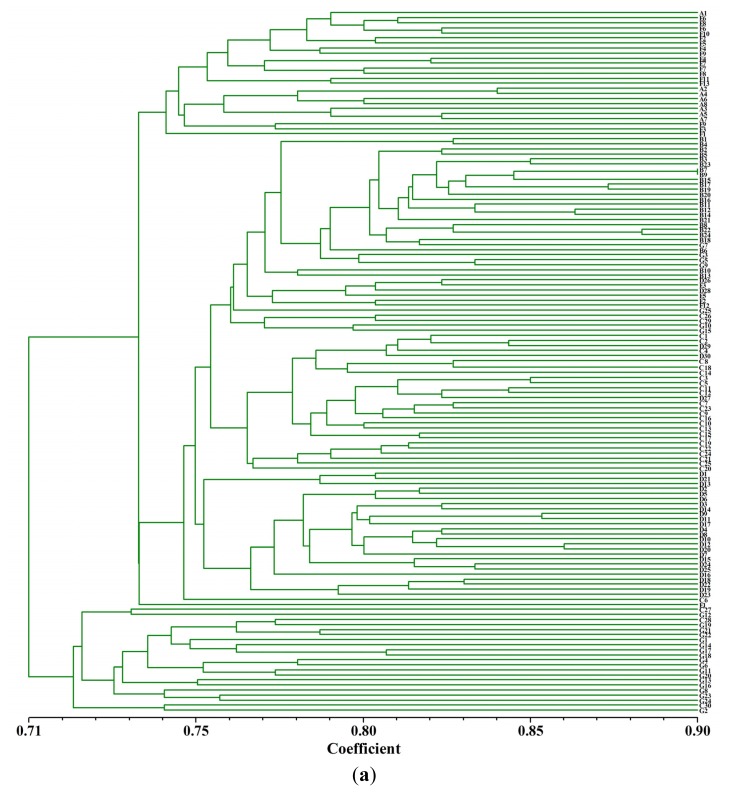

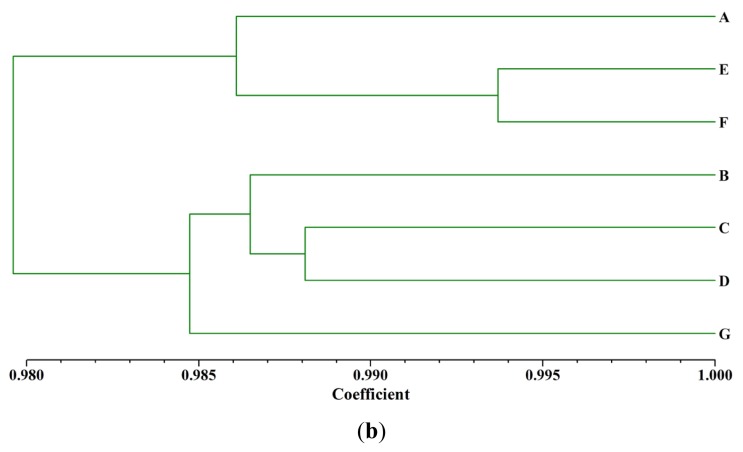
Dendrogram for (**a**) all individuals and (**b**) populations of *T. sutchuenensis* using UPGMA.

The Nei’s genetic distances between pairs of populations were calculated based on the 310 analyzed bands. The values ranged from 0.0064 between population E and F, to 0.0250 between population A and G (Table 5). Furthermore, the dendrogram showed that the individuals and populations were partly mixedly clustered, although being geographically more distant (Figure 2). Consistent to these results, the Mantel test revealed that there was no significant correlation between geographic and genetic distance (*r* = 0.329, *p* = 0.100).

### 2.4. Principal Coordinate Analysis

**Figure 3:**
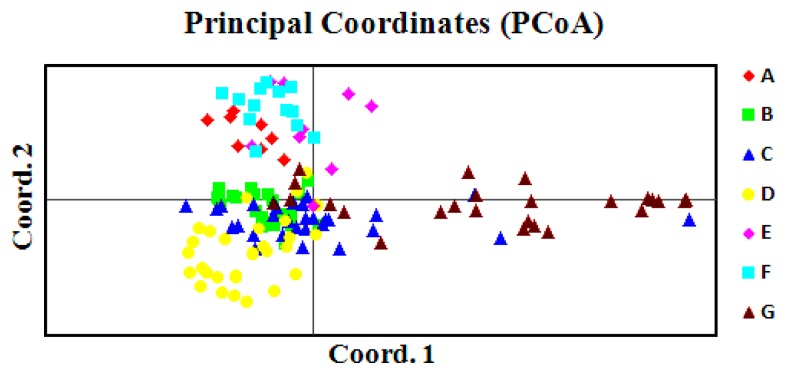
A two-dimensional plot of the Principal Coordinate Analysis (PCoA) of ISSR data showing the clustering of populations of *T. sutchuenensis*. The first and second principal coordinates account for 26.16% and 20.09% of total variation, respectively.

Principal coordinate analysis (PCoA) was performed to provide spatial representation of the relative genetic distances among individuals and to determine the consistency of differentiation among populations defined by the cluster analysis. The first two principal components explained 26.17% and 20.09% of total variation, respectively, and the 63.52% was explained by the first three components (Figure 3). In an agreement with the cluster analysis, individuals from each population formed a separate plot and could be clearly distinguished from those of other populations. The first principal coordinate separated individuals of population A, E and F from the individuals of other four populations. The second principal coordinate separated most individuals of population B, C and D from those of G, which indicated less population-specific identity for population G.

## 3. Discussion

### 3.1. High Genetic Diversity

The ISSR analysis conducted for the *T. sutchuenensis* populations located in North-East Chongqing, China, revealed the presence of an appreciable level of genetic diversity, which was more than that of its relatives *Calocedrus macrolepis* [20], *Taiwania cryptomerioides* [21], *Cupressus gigantea* [22] and *C. chengiana* [23]. This is an expected result for a woody, moderately long-lived, outcrossing and wind-pollinated species [24]. In general, species with small geographic ranges tend to maintain less genetic diversity than geographically widespread species. However, exceptions are not common [19,25,26]. Genetic diversity within populations is influenced by historical factors (e.g., founder effects, bottlenecks, extended time periods with low numbers of individuals and gene flow rate), and thus present-day population sizes may not be a reliable indication of genetic diversity, though population sizes could make positive effects on within population genetic variation [27]. High genetic diversity within small populations can be observed if population size reduction has occurred very recently, especially where that reduction has presented within a generation or two for the species concerned. In such cases the surviving individuals are effectively samples from the larger population that existed before [19,25,28]. It seems to be consistent with witness accounts of local people and records in the present study. During the past decades, the more accessible individuals have been over-exploited for use in home building and for making various household products, which resulting in the rapid reduction of its population size.

### 3.2. Low Genetic Divergence

The overall degree of genetic differentiation, as estimated by *G_ST_*, was 0.102, slightly higher than the mean value (*G_ST_* = 0.073) recorded for 121 woody species examined using allozyme markers [24]. Low level of population differentiation for gymnosperms has also been confirmed by Nybom and Bartish [29] based on an overview of RAPD analysis of plant species, but exceptions were also existed [23,30,31]. Geographic isolation is one major factor influencing genetic differentiation by limiting the amount of gene flow via both pollen and seeds [32]. In theory, gene flow of more than four migrants per generation is sufficient to prevent genetic differentiation between populations due to drift alone [33]. From this standpoint, the level of gene flow estimated (*N_m_* = 4.407) in the present study was enough to rule out the possibility that some differentiation among the populations of *T. sutchuenensis* can be due to isolation. This was consistent with the lack of correlation between genetic and geographic distances (*r* = 0.329, *p* = 0.100), which suggested no significant geographic restriction to gene flow among the populations. The lack of significant correlation between genetic distance and geographical distance also meant that genetic drift played an important role in population differentiation; and when a population was isolated from other populations, the genetic drift became the important factor influencing the genetic structure and increases genetic variation among populations [34]. The results of UPGMA and PCoA also showed no clear geographic trends among natural populations of *T. sutchuenensis*. Therefore, genetic drift may be an explanation for the genetic differentiation among populations.

### 3.3. Implication for Conservation

Thorough understanding of the extent and patterns of genetic diversity in *T. sutchuenensis* is essential for its conservation and exploitation. It is important to understand where efforts should be effectively manage and conserve populations of this species. In the case of *T. sutchuenensis*, AMOVA results shows that each of the seven examined populations currently maintains a relatively high level of within-population genetic diversity. Therefore, the endangered status of this species currently reflects a small number of extant individuals and very poor natural regeneration. Most of the population sizes in the present study are lower than the standard minimum effective population size estimated for plants [35], so maintenance of genetic diversity is not ensured. Conservation measures should therefore focus on establishing large numbers of seedlings, both *in* and *ex situ*, involving many different individuals as parents, to preserve as much as possible of the existing genetic diversity in subsequent generations. Given the current lack of recruitment, establishing seedlings *in situ* will require management and cooperation from local communities. So, *in situ* efforts to conserve remaining habitats need to be combined with *ex situ* research on seed propagation, with a view to establishing a new generation of plants both in cultivation and the wild. In addition, although two local natural protection areas have been established for this species, there still have some other populations outside the protected areas, *i.e.*, population E in the present study. Therefore, a field survey for “new” populations near the distribution range, as well as putting them into protected areas, should also be needed for the species’ protection.

## 4. Experimental Section

### 4.1. Study Species and Population Sampling

*T. sutchuenensis* were found only on bare limestone faces on ridges or steep mountains at North-East Chongqing, China, with different population size. According to the field survey information, seven wild populations of *T. sutchuenensis* with a total of 139 individuals were sampled throughout its distribution range (Table 1; Figure 1). For large populations (*n* > 100), 30 adult individuals were selected with distance of >10 m; while for small populations (*n* < 30), all available adult individuals were sampled. For each sampled individual, fresh leaves (~10 g) were collected and dried in silica gel and stored at −20 °C until subsequent DNA extraction.

### 4.2. DNA Extraction

Total DNA was extracted from silica gel-dried needles of each plant (0.1 g) following the CTAB method [36], as ground to a fine powder in liquid nitrogen and extracted with CTAB extraction buffer [2% (*w*/*v*) CTAB, 20 mM EDTA (Ethylenediamine Tetraacetic Acid), 1.4 mM NaCl, 100 mM Tris-HCl (pH = 8.0), 40 mM b-mercaptoethanol]. The mixture was incubated at 65 °C for 30 min followed by two extractions with chloroform: isoamyl alcohol (24:1). The pellet obtained was washed with 75% ethanol, finally dissolved in TE (Tris-EDTA) buffer [1 mM EDTA (pH = 8.0), 10 mM Tris-HCl (pH = 8.0)]. Quality and concentration of total DNA were verified by spectrophotometry at 260 and 280 nm. DNA samples were stored at −20 °C for ISSR amplification.

### 4.3. ISSR Amplification

A subset of 15 primers (Table 2) chosen from 100 primers (UBC primer set No. 9, Biotechnology Laboratory, University of British Columbia), which yielded bright and discernible bands in two random samples of each population, were finally used for the analysis of all 139 samples. ISSR amplifications were performed in a volume of 25 μL containing 0.4 ng total DNA, 1× PCR buffer, 1.5 mM MgCl_2_, 200 μM dNTPs, 1 μM primer and 0.6 U Taq DNA polymerase (Promega, Madison WI, USA). Amplification reaction was performed in a Gene Amp PCR System 9600 (Perkin Elmer, Foster, CA, USA). The cycle program included an initial 5 min denaturation at 94 °C, followed by 45 cycles of 30 s at 94 °C, 45 s at 50 °C (52 °C for the primer 895#) and 2 min at 72 °C, and 10 min final extension at 72 °C. PCR Products were electrophoresed in 1.6% agarose gel in 0.5× TBE at 100 V for 2 h, stained with ethidium bromide (0.5 μg/mL). The negative control was run by replacing template DNA with ddH_2_O. Gels with amplification fragments were visualized and photographed with DG-III (Beijing Dingguo Co. Ltd., Beijing, China). DL1000 bp ladder (TaKaRa Biotechnology, Dalian, China) was used as DNA molecular weight.

### 4.4. Data Analysis

Only distinct, reproducible, well-resolved fragments were scored as present (1) or absent (0) for each ISSR reaction. The data obtained were combined in a single matrix and genetic coefficients were firstly generated using the program POPGENE v1.32 to describe genetic variation at intra-and inter-population level: the percentage of polymorphic bands (*P*, %), Nei’s gene diversity (*H*), Shannon’s information index (*I*), the observed number of alleles (*N_a_*) and the effective number of alleles (*N_e_*) [37,38]. The genetic structure was further investigated using Nei’s gene diversity statistics, including the total genetic diversity (*H_T_*), genetic diversity within populations (*H_S_*), and the relative magnitude of genetic differentiation among populations (*G_ST_* = (*H_T_* − *H_S_*)/*H_T_*) [37]. An estimate of gene flow among populations (*N_m_*) was computed by the formula of *N_m_* = (1 − *G_ST_*)/2*G_ST_* [39]. The AMOVA (analysis of molecular variance) was performed to describe variance components and their significance levels for variation among individuals within and among the populations, using the program GenAlEx v6.5 [40].

Genetic divergence between populations was investigated using Nei’s unbiased genetic distances and genetic identities [41]. Nei’s unbiased genetic distances were used to construct dendrograms using UPGMA (unweighted pairgroup arithmetic mean-method) [41] in POPGENE v1.32. A principal co-ordinate analysis (PCoA) in GenAlEx 6.5 was employed to examine further the genetic relationships among detected populations on the basis of the same ISSR data. Furthermore, the relationship between geographic and genetic distances was performed with the Mantel test [42].

## 5. Conclusions

In summary, our results indicated the genetic diversity of *T. sutchuenensis* was appreciable both at the population and species level. Low genetic differentiation was found among populations, which may be attributed to limited geographic distance and abundant gene flow. Both UPGMA cluster analysis and PCA supported the grouping of all seven populations into two groups. Based on these findings, strategies are proposed for the conservation of the species.

## Figures and Tables

**Table 1:** Locations of the sampled *T. sutchuenensis* populations and the sampled number (*N*) at each site (Pop = population).

Pop	*N*	Locality	Latitude (° N)	Longitude (° E)	Altitude (m)
A	8	Erchongyan, Kaixian county	31.608	108.712	2290
B	24	Anjiacao, Kaixian county	31.616	108.754	2150
C	30	Wangjiayan, Kaixian county	31.609	108.672	1840
D	30	Yangcun, Kaixian county	31.636	108.811	1770
E	9	Jigongliang, Kaixian county	31.597	108.825	1270
F	13	Geteng, Chengkou county	31.651	108.675	1615
G	25	Longmen, Chengkou county	31.715	108.762	1005

**Table 2:** Sequences of 15 primers successfully used in the ISSR analysis.

Primer	Sequence (5′–3′)	Primer	Sequence (5′–3′)
UBC807	AGA GAG AGA GAG AGA GT	UBC841	GAG AGA GAG AGA GAG AYC
UBC808	AGA GAG AGA GAG AGA GC	UBC842	GAG AGA GAG AGA GAG AYG
UBC809	AGA GAG AGA GAG AGA GG	UBC848	CAC ACA CAC ACA CAC AR*G
UBC810	GAG AGA GAG AGA GAG AT	UBC878	GGA TGG ATG GAT GGA T
UBC811	GAG AGA GAG AGA GAG AC	UBC880	GGA GAG GAG AGG AGA
UBC820	GTG TGT GTG TGT GTG TC	UBC886	VDV CTC TCT CTC TCT CT
UBC826	ACA CAC ACA CAC ACA CC	UBC895	AGA GTT GGT AGC TCT TGA TC
UBC836	AGA GAG AGA GAG AGA GY*A		

* Y: C/T; R: A/G.

**Table 3:** Genetic diversity of *T. sutchuenensis* populations.

	A	B	C	D	E	F	G	Mean	Total
*N_a_*	1.597	1.739	1.913	1.881	1.648	1.690	1.861	1.761	2.000
*N_e_*	1.214	1.180	1.226	1.193	1.215	1.237	1.220	1.212	1.220
*P*	59.7	73.9	91.3	88.1	64.8	69.0	86.1	76.1	100.0
*H*	0.152	0.130	0.161	0.142	0.157	0.163	0.178	0.155	0.165
*I*	0.232	0.180	0.272	0.247	0.245	0.256	0.291	0.252	0.295
*H_T_*									0.167
*H_s_*									0.150
*G_st_*									0.102
*N_m_*									4.407

*N_a_*, observed number of alleles; *N_e_*, effective number of alleles; *P*, percentage of polymorphic loci; *H*, Nei’s gene diversity; *I*, Shannon’s information indices; *H_T_*, total genetic diversity; *H_s_*, genetic diversity within populations; *G_st_*, the relative magnitude of genetic differentiation among populations; *N_m_*, gene flow among populations.

**Table 4:** Analyses of molecular variance (AMOVA) for *T. sutchuenensis* by ISSR.

Source of variation	d.f.*	Sum of squares	Variance component	Percentage of variance	*p* value
Among population	6	783.993	4.938	12.092	<0.01
Within population	132	4738.597	35.898	87.908	<0.01

* *d.f.* = degree of freedom.

**Table 5:** Nei’s genetic distances and geographical distances among populations of *T. sutchuenensis*.

Pop	A	B	C	D	E	F	G
A	----	0.0218	0.0188	0.0210	0.0160	0.0121	0.0250
B	4.12	----	0.0134	0.0138	0.0220	0.0186	0.0167
C	3.78	7.84	----	0.0120	0.0186	0.0183	0.0130
D	9.87	5.77	13.49	----	0.0212	0.0201	0.0163
E	10.79	7.04	14.55	4.48	----	0.0064	0.0202
F	5.92	8.43	4.72	12.97	15.41	----	0.0215
G	12.76	10.93	14.54	9.89	14.33	10.85	----

Above diagonal: genetic distances; below diagonal: geographical distances (km).
